# Women physicians, their social issues & Barriers to their success in Academic Medicine

**DOI:** 10.12669/pjms.38.8.7047

**Published:** 2022

**Authors:** Shaukat Ali Jawaid

**Keywords:** Women Physicians, Social Issues, Leadership role in Academic Medicine

The number of women in medicine is increasing all over the world and Pakistan is no exception. There was a time when only a handful of female students were enrolled in medical colleges of Pakistan, however with the implementation of open merit decided for enrolment a few years ago, the number of female students both in medical and dental colleges has increased and now in almost every medical institution the ratio is between 60-70% girls and 30-40% boys. While their number is increasing, so is the amount of criticism, as most of the females discontinue working for various reasons after marriage.

Women physicians face lot of social issues and we need to address them if we wish to ensure that we do not waste this talent. They also face lot of issues overseas but the initiative foreign countries have taken or plan to take to support female workers in medical field cannot be implemented as such in Pakistan due to cultural and religious norms. Rather we need to modify them as per our requirement, keeping in view the ground realities. Regardless, the role of female physicians in the field of medicine remains unexplored and ignored. There are many barriers to their success in academic medicine as well. In the past and present, we do have women physicians occupying coveted positions, such as Executive Directors of postgraduate medical institutes, Deans, Principals, Pro Vice chancellors of medical universities. Some have even served as Vice chancellors. A large number of them are also heading their respective departments and doing a commendable job. However, female physicians remain under represented in academic leadership positions in Pakistan in contrast to being in majority at the time of enrolment in studies. Hence, it is time that our health planners and those at the helm of affairs urgently address some of the concerns of women physicians and trainees.

While writing in Pulse International recently, I had highlighted some of the issues they face, mentioning the need for flexible training programs to facilitate them so that they can specialize with the provision of safety in work place and comfortable working environment. The facility of transport and Baby Care Centres at work place will encourage married female doctors to continue serving the ailing humanity.^1^

Dr. Aysha Zaheer in an Editorial “The Lost Doctors” published in the current issue has also highlighted the reasons for this lost health workforce. She explains and clears the misconception about various myths related to female physicians, emphasizing that females are equally competent as males in their clinical skills, time management and excel in professionalism.^2^

Historically, medical education for women began in 1847 when Elizabeth Blackwell got admission with one hundred fifty boys in Geneva Medical College. She was ridiculed and bullied by the boys of her class throughout her stay but their behaviour did not discourage Blackwell and she became the first woman in the world to earn a medical degree.^3^-^4^ Over the years, women physicians have become important members of the healthcare teams. Almost 50% of medical students registered for residency in the west are females.

A study by Wietsma in 2014 critically analyses the barriers to success of women physicians and note that they comprised of only 37% full time academic positions in 2012 and increasing to 50% in 2014 is a remarkable improvement in the female work force.^3^ They trace a significant number of women physicians in specialties including paediatrics, obstetrics and gynaecology, internal medicine, radiology, family medicine and basic sciences. The author is of the opinion that for female physicians it is extremely difficult to create a balance between their professional responsibilities, traditional household and parenting duties expected of them. Majority of the women agree that household chores is a barrier to their advancement in medicine. The author further shares his point of view that those who delay in getting married and have children later in life face numerous social issues. “Women’s ability to conceive begins to decline in early 30s while most rapid decline is seen after the age of 37 years”.^3^ In this interesting study about 44% of infertile women physicians were surprised when they came to know about this fact, in spite of their medical background. Even many of them are reported to have expressed regrets not only for delaying conception but also choice of medicine as their professional career.^3^

Some other issues of women physicians which need to be addressed include professional satisfaction, work life balance, parenthood, gender discrimination, academic success and patient satisfaction. A study conducted by Elaine Pelley et al. in 2016 showed that almost 70% of their endocrinology fellows were women They noted lack of interest in this field by male physicians as well as trainees which may result in overall shortage of endocrinologists in future. They anticipated that endocrinology might become a female dominated specialty in the West in the coming days.^5^ On the other hand, the situation is different in many developing countries. It is feared that the prevalence of burnout in women physicians might increase, work life balance challenges are likely to become more important, under representation of women physicians in academic leadership will lead to ignoring their issues. Studies have reported that female physicians are not satisfied with mentoring as well as career advancement opportunities^6^ and they remain under represented in academic medicine.^7^

In developed countries, women physicians are highly represented in certain specialties like endocrinology, rheumatology, geriatrics, but they remain under represented in disciplines like cardiology, pulmonary medicine.^8^ However, in Pakistan most of the women physicians are mostly working in the departments of obstetrics & gynaecology, paediatrics and basic sciences, while many females who are practicing Endocrinology, Radiology and Psychology have their own issues. At the same time acute shortage of male dermatologists in Pakistan is also affecting the quality of patient care as this specialty has now almost been dominated by female and they feel reluctant in examining the male patients.

Yet another study which enrolled 5,704 physicians of which 32% were females in non-surgical specialties found that they were more satisfied with their specialty, patients and colleagues compared to females in surgical specialities. However, they were not satisfied with their limited autonomy. They also complained about discrimination in pay and provision of resources compared to female surgeons. Female physicians attract female patients, particularly those with complex psychological and medical problems. In this study, burnout among female physicians with children who had significant support of spouse and colleagues was remarkably less. Women physicians prefer to work in primary care fields and pay more attention to preventive services, health education, counselling and psychosocial needs of the patients.^9^

Gender discrimination leads to under representation of female physicians in leadership role with the result that their social issues remain neglected. They are paid less attention by the administration and understanding of their working conditions is never a priority. A study from Kuwait revealed that female physicians were well represented in leadership role and this was a very interesting fact. In 2016, almost 50% of leadership role in Kuwait was with female physicians compared to just 40% in 2008. In Mubaak Al-Kabir hospital, female physicians in leadership role increased to 73% in 2016 as compared to 38% in 2008. The specialty they had chosen included Nuclear Medicine, Radiology and Laboratory Medicine. Although the number of female physicians and medical students has progressively increased, significant barriers still restrict their entry into formal leadership role.^10^

However, the situation seems different in Saudi Arabia. A study which assessed the challenges and obstacles encountered by female trainee physicians in Jeddah Saudi Arabia showed that 52% female residents experienced gender discrimination mostly by their supervisors and 40% were regularly harassed. About 53% who agreed to be interviewed were suffering from severe depression, resulting in reconsidering their career in medicine. What was most disturbing was the fact that almost 14% of them thought of committing suicide, four planned to end their life while five had already attempted. Only 6% reported harassment officially to their supervisors. Half of them felt that they were neglected by hospital administration with the result that one fourth were scoring less grades in their studies. This study had concluded that work dis-satisfaction, limited clinical correspondence, higher depression and burnout, stress and dropout rates were all because of gender discrimination. Findings of this study were indeed alarming and a challenge for female trainee physicians.^11^

An online Survey in Pakistan by Malik et al which included one hundred forty six female surgeons of which 67% were trainees showed that 57.5% experienced harassment verbal harassment being most common (64%) but 91.5% did not report it. It also showed that harassment had damaging impact on mental wellbeing of female surgeons. They suggested creating awareness and forming support groups in medical institutions which needs to be addressed urgently.^12^

An effort is being made to find a solution to some of the problems. Some institutions offer faculty reduction in duties, extension of their probationary period or part time employment to those female physicians who are struggling to balance their personal responsibilities at home with professional responsibilities at work place.^13^ But unfortunately no such facility is available in Pakistan. Many women physicians overburdened with home and institutional responsibilities hardly manage time for research and publications. Hence they are also less likely to excel in academic medicine. Lack of quality mentorship is yet another important barrier to their success in this field. Role models in academic medicine not only provide guidance but also have a profound impact on the promotion of their trainees. Female physicians occupying important coveted positions in medical institutions and healthcare facilities attract females of similar calibre, paving way for young female trainees.^13^ In some countries more than 75% of medical schools have designated mentoring for their female students.^7^

Female physicians employed overseas are supporting each other to address the professional inequalities. Faced with gender discrimination, harassment to leadership barrier, they are struggling to survive in this toxic environment. Kim Kelly is the main sponsor of Canadian Medical Association which is educating members on why harassment and gender pay gap is discouraging female physicians into leadership role. She is reported to be working in this field since 1924. An organization named “Canadian Women in Medicine” was launched by Stare Zia after her colleague Dr. Elana Eric was murdered by her husband who himself was a physician. Ever since then, they are engaged in advocacy and intervention when female physicians or trainees face problems both at work and at home.^14^

To ensure that we do not waste the talent of female physicians, we urgently need to address some of the issues and challenges they are facing. It is the duty of not only the parents of the female physicians, but also their in-laws, their senior professional colleagues, health planners, heads of the medical institutions and healthcare facilities to provide safe, secure, comfortable working environment for female physicians. Establishing Baby Care Centres at work place, mentoring facility for female medical students, counselling services and career guidance are some of the initiatives which need to be undertaken on priority basis. Verbal and Sexual harassment of female physicians particularly trainees is very common in Pakistan as well. Most of it goes unreported because of fear of backlash. There is a need to create awareness about it and measures taken to help reporting such incidents ensuring punishment to those involved. At present though some rules exist but they are never implemented in letter and spirit.

Making use of advances in Information Technology and the experience gained during Covid19 pandemic, Telemedicine Centres can be established at major tertiary healthcare facilities and many female physicians can work from home particularly in the field of Radiology, Laboratory Medicine.

Senior Women physicians also need to take interest and come forward to lead this cause. There was time when in Pakistan Army women physicians were not promoted beyond the rank of Brigadier. When one of the senior most Obstetrician & Gynaecologist of her time was refused promotion, she had resigned in protest. Later, the Army changed their rules and regulations and nowadays, they are also promoted to the Rank of Major General. Not only that, even the highest post in Army Medical Corp Surgeon General of Pakistan Army is held by a female. Prof.Khalida Soomro took the initiative of “Go Red” with the result that now in every meeting of Pakistan Cardiac Society and other related sub-specialties there is always a session devoted to Women and Heart Diseases. Such initiatives will definitely go a long way in not only highlighting but also addressing the social issues faced by the women physicians. It will also help them in their fight against gender discrimination and barriers to their advancement in academic medicine. Women should engage themselves in research early in their career and have publications in standard peer reviewed journals with Impact Factor otherwise they will remain at risk of being ignored for leadership role in academic medicine.

## Note:

The author is extremely grateful to Prof. Mariyah Hidayat Prof. of Anatomy at University of Lahore, Prof. Saba Tariq Prof. of Pharmacology and Therapeutics at University Medical & Dental College, Faisalabad, Prof. Nazish Imran, Dept. of Child and Family Psychiatry at KEMU Lahore, Dr. Noori Kiran consultant obstetrician & gynaecologist, medical educationist at Abwa Medical College Faisalabad, Dr. Musarrat Riaz consultant endocrinologist at BIDE, Baqai Medical University for their review, feedback and comments which has greatly improved the quality of this write-up.
